# Quality of life in children at different stages of chronic kidney disease in a developing country

**DOI:** 10.1007/s00467-024-06442-1

**Published:** 2024-08-10

**Authors:** Urapee Chaichana, Julaporn Pooliam, Maturin Jantongsree, Sasitorn Chantaratin, Achra Sumboonnanonda, Anirut Pattaragarn, Suroj Supavekin, Nuntawan Piyaphanee, Kraisoon Lomjansook, Yarnarin Thunsiribuddhichai, Intraparch Tinnabut, Thanaporn Chaiyapak

**Affiliations:** 1 Department of Pediatrics, Faculty of Medicine Siriraj Hospital, Mahidol University, Bangkok, Thailand; 2 Research Development Division, Research Department, Faculty of Medicine Siriraj Hospital, Mahidol University, Bangkok, Thailand; 3 Research Department, Faculty of Medicine, Siriraj Hospital, Mahidol University, Bangkok, Thailand; 4 Division of Psychiatry, Department of Pediatrics, Faculty of Medicine Siriraj Hospital, Mahidol University, Bangkok, Thailand; 5 Division of Nephrology, Department of Pediatrics, Faculty of Medicine Siriraj Hospital, Mahidol University, Bangkok, Thailand; 6 Division of Pediatric Nursing, Department of Nursing, Faculty of Medicine Siriraj Hospital, Mahidol University, Bangkok, Thailand

**Keywords:** Chronic kidney disease, Quality of life, Pediatrics, PedsQL

## Abstract

**Background:**

Children with chronic kidney disease (CKD) require comprehensive assessments, including medical and quality of life (QoL) evaluations. Few studies have been conducted in developing countries.

**Methods:**

This cross-sectional study included 2–18-year-old patients who were categorized into 4 groups: the CKD stage 2–3, stage 4–5, stage 5 with dialysis (D), and kidney transplantation (KT) groups. QoL was measured using the Pediatric Quality of Life Inventory™ (PedsQL™) version 4.0; relationships between different factors and QoL were determined using multivariable linear regression analysis.

**Results:**

Eighty-seven patients (mean age: 13.3 (4.1) years) were included. The self-reported total scores were 77.5 (12.5), 78.9 (11.2), 77.4 (16.2), and 76.1 (10.9) in the stage 2–3, stage 4–5, stage 5D and KT groups, respectively. Parent-reported scores showed a weak-to-moderate correlation with self-reported scores (*r* = 0.12–0.42), with total scores of 71.8 (12.7), 69.5 (14.9), 63.4 (14.8), and 70.8 (18.1) in the stage 2–3, 4–5, 5D and KT groups, respectively. Multivariable linear regression revealed that the parent-reported score in the stage 5D group was 15.92 points lower than that in the stage 2–3 group (*p* = 0.02); the score in the low maternal education group was 10.13 points lower than that in the high maternal education group (*p* = 0.04).

**Conclusions:**

Parent-reported scores showed weak-to-moderate correlation with self-reported scores. Patients with CKD stage 5D and patients with low maternal education had lower QoL. Regular QoL assessment is recommended for patients with advanced CKD and those with socioeconomic vulnerabilities.

**Graphical abstract:**

**Figure:**
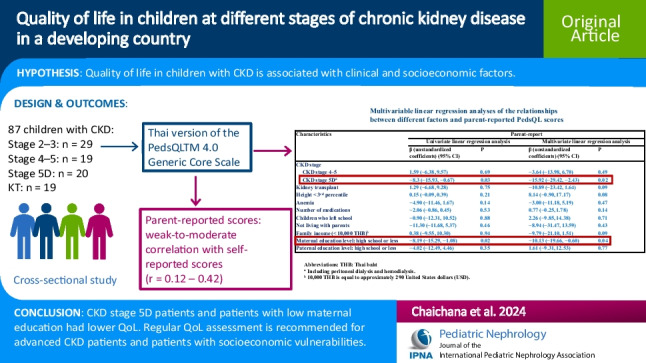
A higher resolution version of the Graphical abstract is available as Supplementary information

**Supplementary Information:**

The online version contains supplementary material available at 10.1007/s00467-024-06442-1.

## Introduction

In the advanced stages of chronic kidney disease (CKD), pediatric patients frequently encounter numerous hurdles that significantly diminish their quality of life (QoL). These challenges include dietary limitations, reliance on multiple medications, daily activity restrictions, barriers to attending school, compromised cognitive function, and an array of complications such as fluid overload and physical transformation [1–3]. Consequently, the care of pediatric CKD patients necessitates a thorough evaluation that encompasses medical and psychosocial dimensions [4]. QoL is a crucial aspect of psychosocial assessment [4, 5]. Although a dedicated instrument exists for evaluating the QoL of pediatric patients with stage 5 CKD [6], there is currently a notable absence of a specific assessment tool for assessing QoL in children at other stages of CKD, including the kidney transplantation (KT) stage. The majority of previous research examining QoL in pediatric CKD patients has predominantly relied on the Pediatric Quality of Life Inventory™ (PedsQL™) [7–12]. Conversely, fewer studies have employed the Greek version of the KIDSCREEN, the Turkish versions of the Kinder Lebensqualität Fragebogen (KINDL®) questionnaire, the Health Utilities Index 3 score, and the KoreaN cohort study for Outcome in patients with Pediatric Chronic Kidney Disease (KNOW-PedCKD) [13–16].

A study conducted in Brazil revealed that pediatric patients in advanced stages of CKD (stages 4–5) and those who underwent KT had significantly lower QoL than healthy children [7]. Similarly, research conducted in the United States (US) indicated that even pediatric patients in early CKD stages (stages 2–3) had lower QoL than healthy children [8]. That study further highlighted that factors such as a low maternal education level, short stature, and a prolonged disease duration negatively impacted QoL [8]. Similarly, a study in Uganda, a developing, low-income country without access to dialysis, revealed that patients in advanced CKD stages reported lower QoL than those in early CKD stages [9]. The majority of QoL studies in pediatric patients with CKD have been conducted in developed, high-income countries, where socioeconomic status and medical care significantly differ from those in developing countries, thereby influencing QoL outcomes. Furthermore, despite being conducted in a developing country, a previous study emphasized the difficulties experienced by patients with CKD stage 5 who lack access to dialysis [9]. This underscores the potential impact of the availability of kidney replacement therapy on long-term survival and QoL outcomes for patients with CKD stage 5 [17, 18]. Although a study on QoL in children with CKD was conducted in Thailand, a developing, upper-middle-income country with access to kidney replacement therapy, including dialysis and KT, it revealed similarly low QoL in children with CKD, with a limited sample size of only 25 patients and a focus solely on adolescents aged 11–18 years [10].

The aim of this study was to evaluate the QoL of pediatric patients at different stages of CKD by considering both total scale scores and individual domain scores. Furthermore, the authors aimed to investigate the potential influence of clinical and socioeconomic factors on QoL outcomes.

## Methods

### Study population

The study population included all patients aged 2–18 years who were diagnosed with CKD stages 2–5D and KT and who were clinically stable for at least 3 months at Siriraj Hospital between September 2022 and November 2023. The exclusion criteria were: (i) acute illness within 30 days, (ii) the loss of a family member within 30 days, due to impact of acute emotional distress on the assessment of QoL, and (iii) the inability to reply to the questionnaire due to illegibility. Patients and their parents could withdraw if they did not wish to participate at any time after enrollment. Patients who met these criteria were informed of the study, and both parental consent and patient assent were obtained.

Clinical data were obtained from electronic patient records and laboratory tests. This study was approved by the Siriraj Hospital Institutional Review Board.

### Quality of life measurement

The Thai version of the PedsQL™ 4.0 Generic Core Scale was utilized to assess QoL in this study. The authors obtained permission to use the Thai version of the PedsQL™ through www.eprovide-mapi-trust.org, with registration number 145835. The questionnaire results were stratified by age group: 2–4, 5–7, 8–12, and 13–18 years. Self-reports were utilized for patients aged 5–18 years, whereas parent reports were employed for patients aged 2–18 years. Only parent-reported data were considered for patients in the 2–4-year-old group. The PedsQL comprises 23 questions across 4 domains: the physical (8 questions), emotional (5 questions), social (5 questions), and school functioning (5 questions) domains. Each item is scored from 0 to 4, and the total scores are converted to a scale of 0 to 100, with higher scores indicating better QoL.

### Clinical definition

The estimated glomerular filtration rate (eGFR) was calculated using the Schwartz formula with an enzymatic method of creatinine measurement. CKD stages 2, 3, 4, and 5 were defined by eGFRs of 60–89, 30–59, 15–29, and < 15 ml/min/1.73 m^2^, respectively. CKD stage 5D was defined as CKD stage 5 treated with either peritoneal dialysis or hemodialysis. KT was defined as receiving a kidney from a donor with a functioning allograft. Laboratory results were collected within a 3-month period of enrollment. According to the World Health Organization (WHO), anemia was defined as serum hemoglobin level < 11.5, < 12, and < 13 g/dL in children aged 6–11.99 years, children aged 12–14.99 years, and male children aged ≥ 15 years, respectively. Hypertension was defined as a blood pressure above the 95th percentile or the use of any antihypertensive medication. The primary caregiver was the main person responsible for the patient. Family income was recorded as the total monthly income, with an exchange rate of 35 Thai baht (THB), approximately equivalent to 1 United States dollar (USD).

### Statistical analysis

Descriptive statistics were used for the general demographic data of the patients. Continuous normally distributed data are presented as the means and standard deviations (SDs), while nonnormally distributed continuous data are presented as medians and interquartile ranges (IQRs). Categorical data are expressed using numbers and percentages. For the comparison of categorical variables, Pearson’s chi-square test was utilized. For continuous variables, either one-way ANOVA or the Kruskal‒Wallis test was employed, chosen based on the distribution of the data. A *p* value < 0.05 was considered to indicate statistical significance.

The mean differences between self-reported and parent-reported scores were analyzed by paired Student’s t tests. The Pearson correlation coefficient (*r*) was calculated and used to analyze QoL scores between self-reports and parent-reports. Very strong, strong, moderate, weak, and negligible correlations were defined as values of 0.90–1.00, 0.70–0.89, 0.40–0.69, 0.10–0.39, and less than 0.10, respectively [19]. Relationships between factors and PedsQL scores were identified using univariable and multivariable linear regression analyses. Any variables that were either significant in the univariable logistic regression analysis (*p* < 0.2) or clinically significant were selected as candidates for multivariable linear regression analysis using the enter method. A *p* value < 0.05 was considered to indicate statistical significance.

All the statistical calculations were performed using IBM SPSS Statistics version 29 (IBM Corp, Armonk, NY, USA).

## Results

### Demographic and clinical characteristics of patients at different stages of CKD

Patients aged 2 to 18 years with CKD stages 2–5D, including KT, were screened for eligibility from September 2022 to November 2023. None of the patients met the exclusion criteria; therefore, all 87 patients were included. Both the patients and their parents completed the PedsQL questionnaire.

A comparison of demographic and clinical characteristics among patients with different stages of CKD is presented in Table 1. Overall, the median age was 14.2 (10.9, 16.6) years, and 57.5% of the patients were male. The common causes of CKD were glomerular disease (28.7%), congenital anomalies of the kidney and urinary tract (CAKUT) (21.8%), and neurogenic bladder (21.8%). Urinary catheters were used in 19 (21.8%) patients due to a neurogenic bladder. The majority of patients (62.1%) were diagnosed with hypertension, and approximately half of the patients met the criteria for anemia. In addition, there were no differences in sex, height, weight, or hypertension among the groups. However, patients in the KT group were older than those in the other groups were (*p* = 0.02). The median duration of KT was 2.9 (0.7, 5.3) years. The majority of patients in the CKD stage 5D and KT groups had glomerular disease, while the causes of CKD in the CKD stage 2–3 and 4–5 groups included both neurogenic bladder and others (*p* = 0.04). Most patients who used urinary catheters were in the CKD stage 2–3 and stage 4–5 groups (*p* = 0.005). Patients in the CKD stage 5D and KT groups were treated with a greater number of medications, with a median of 10 (7.0, 11.0) (*p* < 0.001), and patients in the CKD stage 5D group had a greater incidence of anemia than those in the other groups (*p* < 0.001).

**Table 1:** Comparison of demographic and clinical characteristics of patients at different stages of chronic kidney disease

Characteristics	CKD stage 2–3, *n* (%) *n* = 29	CKD stage 4–5, *n* (%) *n* = 19	CKD stage 5 D^a^, *n* (%) *n* = 20	Kidney transplant, *n* (%) *n* = 19	Total *n* = 87	*P*
Male	17 (58.6)	10 (52.6)	12 (60)	11 (57.9)	50 (57.5)	0.98
Age, years (IQR)	12.8 (8.5, 16.6)	13.0 (7.6, 16.8)	15.9 (12.9, 16.8)	15.8 (13.8, 17.1)	14.2 (10.9, 16.6)	0.02
Cause of CKD						
Glomerular disease	4 (13.8)	4 (21.1)	9 (45)	8 (42.1)	25 (28.7)	0.04
CAKUT	5 (17.2)	5 (26.3)	6 (30)	3 (15.8)	19 (21.8)	
Neurogenic bladder	9 (31)	5 (26.3)	1 (5)	4 (21.1)	19 (21.8)	
Unknown	0 (0)	0 (0)	2 (10)	2 (10.5)	4 (4.6)	
Others	11 (37.9)	5 (26.3)	2 (10)	2 (10.5)	20 (23)	
Height, cm (SD)	141.0 (23.2)	138.8 (17.7)	147.8 (20.5)	148.5 (14.2)	143.7 (19.8)	0.30
Weight, kg (SD)	41.8 (20.7)	36.4 (12.4)	50.7 (29.3)	48.1 (12.6)	44.0 (20.6)	0.12
Urinary catheter use	9 (31)	8 (42.1)	2 (10)	0 (0)	19 (21.8)	0.005
Number of medications, median (IQR)	2 (0, 4.0)	6 (3.0, 8.0)	12 (9.3, 15.0)	10 (7.0, 11.0)	7 (0, 21.0)	< 0.001
Hypertension	14 (48.3)	10 (52.6)	17 (85)	13 (68.4)	54 (62.1)	0.16
Anemia	9 (31)	11 (57.9)	19 (95)	10 (52.6)	49 (56.3)	< 0.001

*CAKUT* congenital anomalies of the kidney and urinary tract, *CKD* chronic kidney disease, *cm* centimeter, *D* dialysis, *IQR* interquartile range, *kg* kilogram, *SD* standard deviation

^a^Including peritoneal dialysis and hemodialysis

### Socioeconomic characteristics of patients at different stages of chronic kidney disease

The socioeconomic characteristics are summarized in Table 2, and there were no differences in socioeconomic characteristics among the groups. The majority of patients received education within the system (80.5%), which included school, college, and university. All of the patients were covered under the universal health coverage. Approximately half of the patients (46%) resided in rural communities, with the majority living in their home (77%). Regarding family characteristics, 77% belonged to complete families, with a median of 4.5 (2.0, 9.0) family members. The median monthly family income per household was 20,000 (15,000, 40,000) THB or approximately 571 (428, 1,142) USD. The main caregivers were predominantly parents (92.0%), which was consistent across all groups. Most mothers (70.6%) and fathers (81.2%) had a high school education or lower. Additionally, the majority of the parents were in good health (73.3% of mothers and 68.4% of fathers). The measurement of the parents’ health was conducted subjectively.

**Table 2:** Comparison of socioeconomic characteristics of patients at different stages of chronic kidney disease

Characteristics	CKD stage 2–3, *n* (%) *n* = 29	CKD stage 4–5, *n* (%) *n* = 19	CKD stage 5 D^a^, *n* (%) *n* = 20	Kidney transplant, *n* (%) *n* = 19	Total *n* = 87	*P*
Type of education						
School/college/university	25 (86.2)	17 (89.5)	13 (65)	15 (78.9)	70 (80.5)	0.69
Informal education	0 (0)	0 (0)	4 (20)	3 (15.8)	7 (8)	
Children who left school	2 (6.9)	2 (10)	3 (15)	1 (5.3)	8 (9.2)	
Underage	2 (6.9)	0 (0)	0 (0)	0 (0)	2 (2.3)	
Universal Coverage Scheme	29 (100)	19 (100)	20 (100)	19 (100)	87 (100)	NA
Lives in a rural area	11 (37.9)	7 (36.8)	9 (45)	13 (68.4)	40 (46)	0.16
Type of residence						
House/townhouse	26 (89.7)	19 (100)	20 (100)	17 (89.5)	82 (94.3)	0.23
Apartment/flat/condominium	3 (10.3)	0 (0)	0 (0)	2 (10.5)	5 (5.7)	
House owner	21 (72.4)	15 (78.9)	16 (80)	15 (78.9)	67 (77)	0.91
Family structure						
Full	21 (72.4)	15 (78.9)	16 (80)	15 (78.9)	67 (77)	0.65
Single parent	6 (20.7)	4 (21.1)	2 (10)	4 (21.1)	16 (18.4)	
Not living with parents	1 (3.4)	0 (0)	2 (10)	0 (0)	3 (3.4)	
Number of family members, (IQR)	4 (4.0, 6.0)	4 (3.0, 4.0)	4.5 (4.0, 5.8)	4 (3.0, 5.0)	4.5 (2.0, 9.0)	0.07
Siblings	21 (72.4)	11 (57.9)	17 (85)	10 (52.6)	59 (67.8)	0.12
Family income, 10,000 THB^b^ (IQR)	2 (1.5, 4.0)	2 (1.0, 4.0)	2 (1.2, 3.8)	2 (1.0, 5.0)	2 (1.5, 4.0)	0.97
Main caregiver						
Parents	26 (89.7)	17 (89.5)	18 (90)	19 (100)	80 (92)	0.55
Grandparents/others	2 (6.9)	1 (5.3)	0 (0)	0 (0)	3 (3.4)	
Others	1 (3.4)	1 (5.3)	2 (10)	0 (0)	4 (4.6)	
Maternal data (*n* = 85)						
Age, years (SD)	42.9 (7.8)	39.0 (7.3)	41.3 (6.1)	43.0 (7.6)	41.8 (7.3)	0.31
Highest education						
High school or lower	20 (71.4)	14 (73.6)	15 (78.9)	11 (57.9)	60 (70.6)	0.49
Bachelor’s degree or higher	8 (28.6)	5 (26.4)	4 (21.1)	8 (42.1)	25 (29.4)	
Healthy	19 (67.9)	15 (78.9)	13 (65)	16 (84.2)	63 (73.3)	0.46
Paternal data (*n* = 85)						
Age, years (SD)	44.7 (7.8)	44.5 (10.8)	43.9 (8.9)	45.9 (8.5)	44.8 (8.8)	0.68
Highest education						
High school or lower	25 (89.3)	13 (72.2)	18 (90)	13 (68.4)	69 (81.2)	0.69
Bachelor’s degree or higher	3 (10.7)	5 (27.8)	2 (10)	6 (31.6)	16 (18.8)	
Healthy	18 (69.2)	11 (64.7)	15 (83.3)	10 (55.6)	54 (68.4)	0.34

*CKD* chronic kidney disease, *D* dialysis, *IQR* interquartile range, *NA* not applicable, *THB* thai baht

^a^Including peritoneal dialysis and hemodialysis

^b^10,000 THB is equal to approximately 350 United States dollars (USD)

### PedsQL scores and the correlation between self-reported and parent-reported scores among the different CKD stage groups

Table 3 presents PedsQL score correlations between self-reported scores and parent-reported scores among the different CKD stage groups. The analysis included 83 pairs of reports, excluding patients aged 2–4 years due to exclusive parent-reported responses in this age group (2 patients in the CKD stage 2–3, and 1 patient in the CKD stage 4–5). Weak-to-moderate correlations were observed between self-reported and parent-reported total PedsQL scores in the CKD stage 2–3 (*r* = 0.12), stage 4–5 (*r* = 0.42), stage 5D (*r* = 0.41), and KT groups (*r* = 0.42). Additionally, the correlations between self-reported and parent-reported scores on various PedsQL domains ranged from negligible to moderate among the different CKD stage groups (*r* = -0.76 to 0.53).

**Table 3:** PedsQL scores and the correlation between self-reported and parent-reported scores among the different CKD stage groups

Variables	CKD stage 2–3 (*n* = 27) mean (SD)			CKD stage 4–5 (*n* = 18) mean (SD)			CKD stage 5 D^a^ (*n* = 20) mean (SD)			Kidney transplant (*n* = 19) mean (SD)			Total (*n* = 84) mean (SD)		
	Self-reports	Parent-reports	*r*	Self-reports	Parent-reports	*r*	Self-reports	Parent-reports	*r*	Self-reports	Parent-reports	*r*	Self-report	Parent-reports	*r*
Physical functioning	82.2 (12.9)	71.9 (18.4)	0.31	79.9 (12.7)	70.1 (21.4)	0.14	77.3 (15.2)^*^	63.1 (16.9)	0.45	75.8 (18.1)	70.9 (22.6)	0.41	79.1 (13.6)^*^	69.8 (19.8)	0.33
Emotional functioning	72.8 (18.9)^*^	73.5 (14.7)	0.45	75.0 (15.9)	69.4 (12.9)	0.13	77.8 (21.9)^*^	66.8 (19.2)	0.42	74.5 (20.1)	71.6 (20.3)	0.43	74.8 (19.1)	71.1 (16.8)	0.37
Social functioning	83.3 (15.9)	77.2 (17.3)	0.12	87.8 (14.3)^*^	73.6 (18.2)	0.46	88.0 (17.9)^*^	70.0 (17.4)	0.47	79.4 (13.7)	79.2 (19.0)	0.32	84.5 (15.7)^*^	75.9 (17.9)	0.27
School functioning	71.7 (14.6)	64.4 (17.4)	− 0.76	73.3 (12.7)^*^	65.0 (19.1)	0.53	66.5 (20.1)^*^	54.2 (20.5)	0.40	74.7 (12.6)^*^	61.6 (20.7)	0.03	71.5 (15.4)^*^	62.5 (19.9)	0.24
Total score	77.5 (12.5)	71.8 (12.7)	0.12	78.9 (11.2)^*^	69.5 (14.9)	0.42	77.4 (16.2)^*^	63.4 (14.8)	0.41	76.1 (10.9)	70.8 (18.1)	0.42	77.5 (12.7)^*^	69.8 (15.4)	0.31

*CKD* chronic kidney disease, *D* dialysis, *PedsQL* pediatric quality of life inventory, *SD* standard deviation, *r*: correlation

^a^Including peritoneal dialysis and hemodialysis

*Difference between self and parent reports; *p* < 0.05

In all CKD stage groups and PedsQL domains, self-reported scores consistently exceeded parent-reported score. Overall, self-reported physical, social, school, and total scores were significantly greater than parent-reported scores (*p* < 0.05). In the CKD stage 2–3 group, self-reported scores were significantly greater for emotional functioning than parent-reported scores (*p* < 0.05). In the CKD stage 4–5 group, self-reported social, school functioning, and total scores were significantly greater (*p* < 0.05) than parent-reported scores. For CKD stage 5D patients, self-reported scores were significantly greater for all dimensions, as well as the total score (*p* < 0.05) compared to parent-reported scores. Similarly, in the KT group, self-reported scores were significantly greater for school functioning than parent-reported scores (*p* < 0.05).

The self-reported total scores were 77.5 (12.5), 78.9 (11.2), 77.4 (16.2), and 76.1 (10.9) in the CKD stage 2–3, stage 4–5, stage 5D and KT groups, respectively. Parent-reported total scores showed weak-to-moderate correlations with self-reported scores (*r* = 0.12–0.42), with scores of 71.8 (12.7), 69.5 (14.9), 63.4 (14.8), and 70.8 (18.1) in the CKD stage 2–3, stage 4–5, stage 5D and KT groups, respectively. While self-reported PedsQL total scores and domain scores did not significantly differ among the CKD stage groups, parent-reported PedsQL scores revealed notable distinctions. According to parent reports, the CKD stage 5D group consistently had the lowest scores across all domains compared to other groups, but this difference was not statistically significant. For instance, for physical functioning the parent-reported scores were 63.1 (16.9) for the CKD stage 5D group, 71.9 (18.4) for the CKD stage 2–3 group, 70.1 (24) for the CKD stage 4–5 group, and 70.9 (22.6) for the KT group (*p* = 0.38). Similar patterns were observed in the emotional (*p* = 0.54), social (*p* = 0.40), and school functioning domains (*p* = 0.15), where the CKD stage 5D group consistently showed the lowest scores among the groups.

### The relationships between different factors and self-reported and parent-reported PedsQL scores

A univariable linear regression analysis was performed to explore the relationships between different factors and PedsQL scores, as presented in Table 4. The univariable linear regression analysis of self-reported scores revealed no statistically significant associations. In contrast, the univariable linear regression analysis of parent-reported scores identified CKD stage 5D (*β*: –8.3, 95% CI: (–15.93, –0.67, *p* = 0.03) and a low maternal education level (*β*: –8.19, –15.29, –1.08, *p* = 0.03) as potential factors associated with lower PedsQL scores. Other risk factors were not statistically significant.

**Table 4:** Univariable linear regression analyses of the relationships between different factors and self- and parent-reported PedsQL scores

Characteristics	Self-reported scores		Parent-reported scores	
	*β* (unstandardized coefficients) (95% CI)	*P*	*β* (unstandardized coefficients) (95% CI)	*P*
Clinical characteristics				
Age	0.49 (− 0.27, 1.25)	0.21	− 0.13 (− 0.95, 0.69)	0.75
Female	− 1.38 (− 9.12, 2)	0.21	0.34 (− 6.33, 7.01)	0.92
CKD stage	− 0.11 (− 6.63, 6.41)	0.97	− 8.30 (− 15.93, − 0.67)	
CKD stage 2–3	Reference		Reference	
CKD stage 4–5	1.51 (− 6.31, 9.32)	0.70	1.59 (− 6.38, 9.57)	0.69
CKD stage 5 D^a^	− 0.09 (− 7.67, 7.49)	0.98	− 8.3 (− 15.93, − 0.67)	0.03
Kidney transplant	− 1.36 (− 9.06, 6.33)	0.73	1.29 (− 6.68, 9.28)	0.75
Height < 3rd percentile	0.93 (− 5.58, 7.44)	0.78	0.15 (− 0.09, 0.39)	0.21
Weight < 3rd percentile	1.23 (− 6.44, 8.90)	0.75	− 3.49 (− 12.71, 5.73)	0.45
Cause of CKD				
CAKUT	Reference		Reference	
Glomerular disease	− 3.42 (− 11.33, 4.49)	0.39	0.80 (− 8.24, 9.85)	0.86
Neurogenic bladder	− 0.48 (− 9.14, 8.17)	0.91	9.78 (0.14, 19.42)	0.047
Unknown	− 0.27 (− 8.25, 7.71)	0.95	− 2.87 (− 11.99, 6.26)	0.53
Urinary catheter use	3.38 (− 3.49, 10.25)	0.33	4.33 (− 3.59, 12.26)	0.28
Number of medications	− 0.23 (0.57, 5.20)	0.93	− 0.21 (− 0.86, 0.45)	0.53
Hypertension	0.67 (− 4.81, 6.18)	0.81	0.81 (− 5.75, 7.38)	0.81
Anemia	− 0.76 (− 6.39, 4.87)	0.79	− 4.90 (− 11.46, 1.67)	0.14
Socioeconomic characteristics				
Children who left school	4.31 (− 5.69, 14.31)	0.39	− 0.90 (− 12.31, 10.52)	0.88
Live in rural area	1.70 (− 3.86, 7.25)	0.55	− 2.69 (− 9.28, 3.90)	0.42
Not living with parents	− 1.37 (− 17.45, 14.72)	0.87	− 11.30 (− 11.68, 5.37)	0.46
Number of family members	− 0.17 (− 2.01, 1.69)	0.86	0.29 (− 1.95, 2.52)	0.80
Family income > 10,000 THB^b^	1.07 (− 7.15, 9.29)	0.80	0.38 (− 10.30, 9.55)	0.94
Maternal education level: high school or less	2.41 (− 3.77, 8.59)	0.44	− 8.19 (− 15.29, − 1.08)	0.03
Paternal education level: high school or less	5.73 (− 1.45, 12.92)	0.12	− 4.02 (− 12.49, 4.46)	0.35
Main caregiver not parents; grandparents	1.65 (− 8.39, 11.69)	0.74	− 0.67 (− 12.80, 11.44)	0.91

*95% CI* 95% confidence interval, *CKD* chronic kidney disease, *D* dialysis, *PedsQL* pediatric quality of life inventory, *THB* thai baht, *β* unstandardized coefficients

^a^Including peritoneal dialysis and hemodialysis

^b^10,000 THB is equal to approximately 350 United States dollars (USD)

The *β* coefficient indicates the difference in the domain z score per 1-point increase in the PedsQL score

A total of 87 patients were included in this study; hence, 9 factors could be selected as candidates for the multivariate linear regression analysis model (with 10 patients per factor). The results of the multivariate linear regression analysis (Table 5) for parent reports identified CKD stage (*p* = 0.03 in CKD stage 5D patients), height < 3rd percentile (*p* = 0.21, clinically significant), anemia (*p* = 0.14), number of medications (*p* = 0.53, clinically significant), leaving school (*p* = 0.88, clinically significant), not living with parents (*p* = 0.46, clinically significant), low family income (*p* = 0.94, clinically significant), a low maternal education level (*p* = 0.02), and a low paternal education (*p* = 0.35, clinically significant) as factors for the final model built using the enter method. Notably, CKD stage 5D patients and patients whose mothers had low education levels were significantly associated with PedsQL scores that were 15.92 points (*p* = 0.02) and 10.13 points (*p* = 0.04) lower, respectively, than those of patients in the early stages of CKD and whose mothers had high education levels.

**Table 5:** Multivariable linear regression analyses of the relationships between different factors and self- and parent-reported PedsQL scores

Characteristics	Child self-report				Parent-report			
	Univariate linear regression analysis		Multivariate linear regression analysis		Univariate linear regression analysis		Multivariate linear regression analysis	
	*β* (unstandardized coefficients) (95% CI)	*P*	*β* (unstandardized coefficients) (95% CI)	*P*	*β* (unstandardized coefficients) (95% CI)	*P*	*β* (unstandardized coefficients) (95% CI)	*P*
CKD stage								
CKD stage 4–5	1.51 (− 6.31, 9.32)	0.70	2.97 (− 6.66, 12.60)	0.54	1.59 (− 6.38, 9.57)	0.69	− 3.64 (− 13.98, 6.70)	0.49
CKD stage 5 D^a^	− 0.09 (− 7.67, 7.49)	0.98	0.97 (− 11.32, 13.25)	0.88	− 8.3 (− 15.93, − 0.67)	0.03	− 15.92 (− 29.42, − 2.43)	0.02
Kidney transplant	− 1.36 (− 9.06, 6.33)	0.73	0.54 (− 10.84, 11.91)	0.93	1.29 (− 6.68, 9.28)	0.75	− 10.89 (− 23.42, 1.64)	0.09
Height < 3rd percentile	0.93 (− 5.58, 7.44)	0.78	0.63 (− 7.50, 8.75)	0.88	0.15 (− 0.09, 0.39)	0.21	8.14 (− 0.90, 17.17)	0.08
Anemia	− 0.76 (− 6.39, 4.87)	0.79	− 1.11 (− 8.59, 6.37)	0.77	− 4.90 (− 11.46, 1.67)	0.14	− 3.00 (− 11.18, 5.19)	0.47
Number of medications	− 0.23 (− 0.57, 0.53)	0.93	− 0.01 (− 0.93, 0.91)	0.98	− 2.06 (− 0.86, 0.45)	0.53	0.77 (− 0.25, 1.78)	0.14
Children who left school	4.31 (− 5.69, 14.31)	0.39	3.50 (− 8.49, 15.50)	0.56	− 0.90 (− 12.31, 10.52)	0.88	2.26 (− 9.85, 14.38)	0.71
Not living with parents	− 1.37 (− 17.45, 14.72)	0.87	− 4.91 (− 25.19, 15.37)	0.63	− 11.30 (− 11.68, 5.37)	0.46	− 8.94 (− 31.47, 13.59)	0.43
Family income (< 10,000 THB)^b^	1.07 (− 7.15, 9.29)	0.80	1.45 (− 8.74, 11.64)	0.78	0.38 (− 9.55, 10.30)	0.94	− 9.79 (− 21.10, 1.51)	0.09
Maternal education level: high school or less	2.41 (− 3.77, 8.59)	0.44	− 0.86 (− 9.54, 7.82)	0.84	− 8.19 (− 15.29, − 1.08)	0.02	− 10.13 (− 19.66, − 0.60)	0.04
Paternal education level: high school or less	5.73 (− 1.45, 12.92)	0.12	6.69 (− 3.20, 16.58)	0.18	− 4.02 (− 12.49, 4.46)	0.35	1.61 (− 9.31, 12.53)	0.77

*95% CI* 95% confidence interval, *CKD* chronic kidney disease, *D* dialysis, *PedsQL* pediatric quality of life inventory, *THB* Thai baht, *β* unstandardized coefficients

^a^Including peritoneal dialysis and hemodialysis

^b^10,000 THB is equal to approximately 350 United States dollars (USD)

The *β* coefficient indicates the difference in the domain z score per 1-point increase in the PedsQL score

## Discussion

This study aimed to assess QoL scores in children at different stages of CKD in a developing country and to identify the potential impact of clinical and socioeconomic factors on QoL outcomes. Parent-reported scores consistently lagged behind self-reported scores at all CKD stages, showing a weak-to-moderate correlation (*r* = 0.12–0.42). Additionally, in parent-reported assessments, the score of the CKD stage 5D group was significantly lower than that of the early-stage CKD group (by 15.92 points (*p* = 0.02), while the low maternal education group score was 10.13 points lower than that of the high maternal education group (*p* = 0.04).

The patients included in this study had clinical characteristics similar to those of the populations of other studies on QoL in pediatric patients with CKD, including age, sex, etiology of CKD, and anemia [7–9, 11, 12]. This comparison underscores the representativeness of our sample and supports the generalizability of our findings. However, the socioeconomic status in this study was lower than that observed in the multicenter study from the CKiD cohort, which was conducted in developed countries [8]. In contrast to studies conducted in developed countries where the maternal education level predominantly exceeded high school levels [8], the majority of mothers in this study had a high school education or lower, similar to findings in another study conducted in a developing country [9]. The main caregivers in this study were parents, and most of the children lived with a full family structure, similar to the findings of other studies [7, 9, 11].

Since CKD is a chronic illness, many guidelines recommend maximizing QoL and incorporating QoL assessments as part of regular patient evaluations [2, 20, 21]. Previous pediatric research has shown that pediatric patients with CKD tend to have lower QoL than healthy children [7–12]. Similarly, this study reaffirms these observations, demonstrating that pediatric patients at all stages of CKD exhibited reduced QoL. Although we did not perform statistical analysis, an observational comparison indicates that the QoL scores in our CKD cohort appear lower than those of healthy Thai children, as studied by Sritipsukho et al. [22]. Additionally, Lopes et al. [7] demonstrated that individuals with CKD stage 5D had lower total, physical, emotional, and school domain scores than KT patients did. Similarly, this study underscores this disparity, revealing that patients with CKD stage 5D, as reported by parents, had lower QoL scores across all domains than patients in other CKD groups.

In a previous study [8], no significant difference was found between parent- and self-reported PedsQL scores, with some studies suggesting higher scores from parent-reported assessments than from self-reported assessments [7, 9]. Conversely, other studies indicated lower PedsQL scores in parent-reported assessments than in self-reported assessments [10, 12]. According to studies in developed countries [7, 8, 12], there were inconsistent results regarding the correlation between parent-reported and self-reported scores. This study revealed a weak-to-moderate correlation between parent-reported and self-reported scores. Parent-reported assessments consistently revealed lower PedsQL scores than self-reported assessments across all stages of CKD and domains, which was particularly notable in patients in advanced stages of CKD (stage 4–5D). This discrepancy may arise because parents often consider the overall well-being of their children, whereas the children themselves may not perceive certain issues from their childlike perspective. Additionally, the heightened caregiving responsibilities undertaken by parents for pediatric patients with CKD, especially in advanced stages, can lead to elevated stress levels and increased financial burdens for caregivers [23]. Moreover, parents may be more attuned to subtle changes in their child’s condition and the long-term implications of CKD, leading them to report lower QoL scores. This phenomenon is supported by research indicating that parents’ stress, anxiety, and concern for their child’s future significantly influence their perception of the child’s QoL [24–27].

Previous studies of pediatric patients have revealed both physical and mental burdens in those receiving dialysis [28, 29]. Similarly, this study demonstrated that patients with CKD stage 5D had significantly lower parent-reported PedsQL scores (by 15.92 points) than patients with CKD in the early stages. One potential explanation is that patients with CKD stage 5 typically undergo complex medication regimens with multiple doses throughout the day, experience physical weakness, including failure to thrive, face dietary restrictions, often miss school due to medical appointments or hospital admissions, rely on hemodialysis or peritoneal dialysis machines for treatment, and may experience feelings of depression [2]. In this study, a significant association was observed between low maternal education levels and parent-reported PedsQL scores: the parent-reported PedsQL score was 10.13 points lower in the low maternal education group than in the high maternal education group. These findings are in line with a previous study by Gerson et al. [8], which identified a relationship between low maternal education levels and QoL in patients with mild to moderate CKD. Moreover, a low maternal education level has been shown to impact children’s QoL across various diseases, including thalassemia, cancer, type 1 diabetes mellitus, asthma, and overweight/obesity [30–32]. One potential explanation is that a lower maternal education level is often correlated with a lower household income level, resulting in limited access to nutritious food, safe housing, and educational opportunities. Additionally, a low maternal education level is often associated with increased stress and limited knowledge and skills to provide support and guidance to children. These factors can collectively impact the QoL of a child and the parent’s perception of the child’s QoL. Previous studies have revealed that low parental socioeconomic status, including low parental education, affects a child’s health and QoL [31–33]. Additional potential factors associated with QoL in patients with CKD, as reported in previous pediatric studies [8], include younger age and short stature. However, these variables were not found to be statistically significant in this study.

This study has several strengths. First, this study encompassed a relatively large population compared to a previous study conducted in Thailand [10] that utilized the same PedsQL instrument. Second, the population encompassed a wide range of ages, from 2 to 18 years, rather than being limited to a specific age group. Third, the generalizability of this study to all children with CKD in developing countries is notable. While conducted in a tertiary center, approximately half of the patients resided in rural areas and were referred from local hospitals across all regions of Thailand. Fourth, this study examined the relationships between different factors and QoL. Based on a review of the literature, only one previous study conducted in a developed country investigated this variable [8]. This study revealed that CKD stage 5D and low maternal education levels were associated with low QoL, and these results expand on those in the literature. Our study also has several limitations. First, this was a cross-sectional study assessing QoL in patients with CKD. However, QoL is subject to change over time due to various factors, such as alterations in health status, socioeconomic circumstances, and life events. Therefore, a longitudinal analysis could provide more comprehensive information regarding the dynamic nature of QoL in this population, as demonstrated by previous longitudinal studies of QoL in pediatric patients with CKD [34]. Second, this study employed the PedsQL, relying on information provided by the patients and their parents. As a result, the assessment of school functioning may not be entirely accurate due to the absence of first-hand data during school hours. Therefore, supplementing the data with information obtained from teachers regarding learning issues would contribute to the comprehensiveness of the dataset. Third, there was no comparison of QoL between the patients with CKD and healthy controls in this study. The study period coincided with the COVID-19 outbreak in Thailand, which led to online schooling and increased use of telemedicine, making it difficult to recruit healthy controls for a direct comparison. Although we did not perform a statistical analysis, an observational comparison suggests that the QoL scores in our CKD cohort are lower than those of healthy Thai children, as reported by Sritipsukho et al. [22]. Therefore, there is a need for additional prospective longitudinal studies to assess QoL among patients at various stages of CKD while incorporating teacher assessments of school functioning. Additionally, comparing these findings with those of a healthy control group would further validate the outcomes of this study.

In conclusion, this study demonstrated a significant association between low QoL and CKD stage 5, as well as between low QoL and low maternal education levels. We recommend regular QoL assessment for children with advanced CKD and those at socioeconomic risk.

## Supplementary Information

Below is the link to the electronic supplementary material.

Graphical abstract (PPTX 84 KB)

## Data Availability

Not applicable.
