# Supplementary material for: Quality of life in children at different stages of chronic kidney disease in a developing country
Source: Pediatr Nephrol. 2024 Aug 10;40(1):177–87. doi: 10.1007/s00467-024-06442-1 (PMC11584483; doi:10.1007/s00467-024-06442-1)
Supplement: Supplementary file 1 — Graphical abstract (PPTX 84 KB) [file 467_2024_6442_MOESM1_ESM.pptx]

## Slide 1
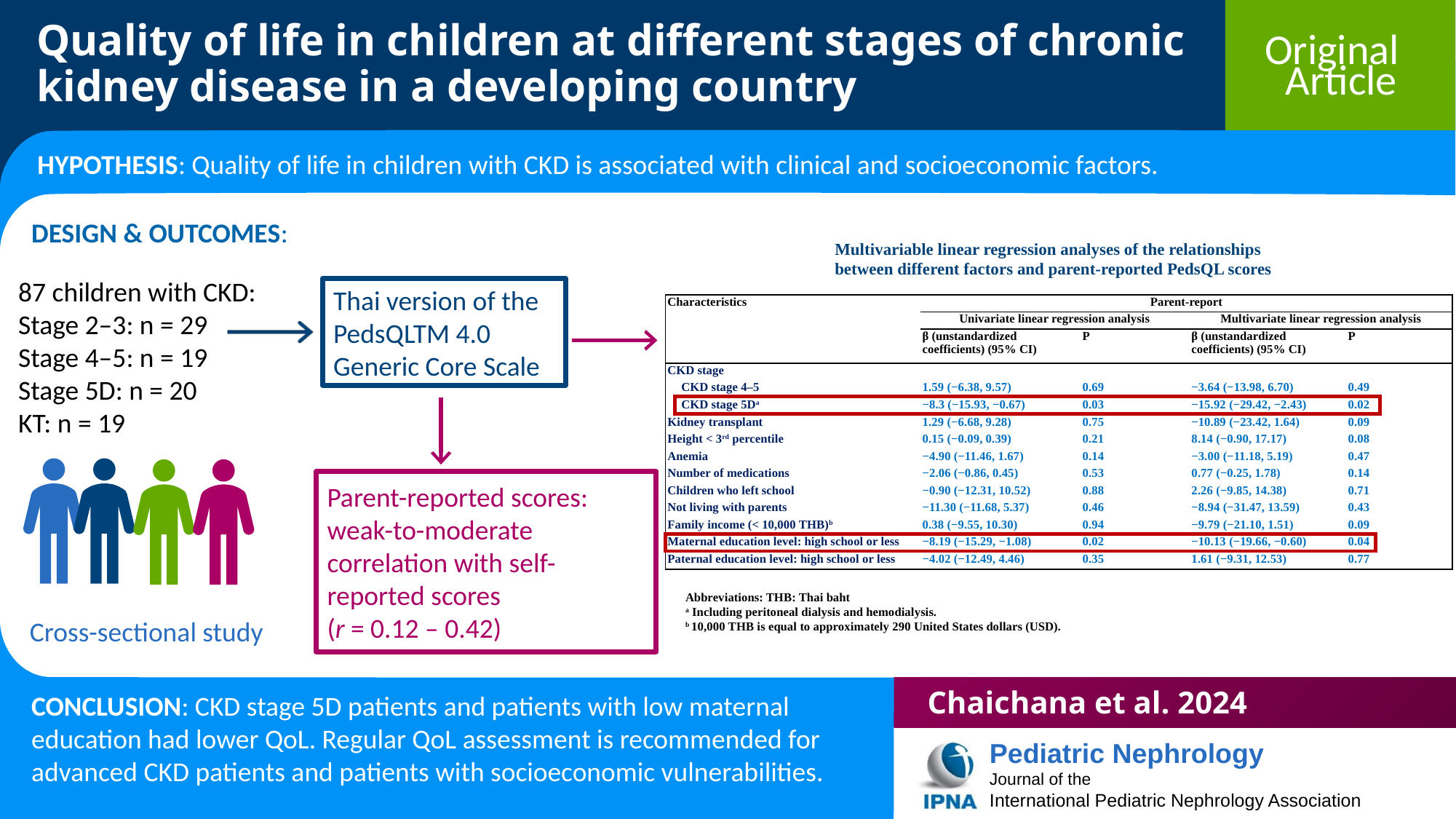

Quality of life in children at different stages of chronic kidney disease in a developing country
HYPOTHESIS: Quality of life in children with CKD is associated with clinical and socioeconomic factors.
DESIGN & OUTCOMES:
Multivariable linear regression analyses of the relationships
between different factors and parent-reported PedsQL scores
Thai version of the PedsQLTM 4.0 Generic Core Scale
87 children with CKD:
Stage 2–3: n = 29
Stage 4–5: n = 19
Stage 5D: n = 20
KT: n = 19
| Characteristics | Parent-report | | | |
| --- | --- | --- | --- | --- |
| | Univariate linear regression analysis | | Multivariate linear regression analysis | |
| | β (unstandardized coefficients) (95% CI) | P | β (unstandardized coefficients) (95% CI) | P |
| CKD stage | | | | |
| CKD stage 4–5 | 1.59 (−6.38, 9.57) | 0.69 | −3.64 (−13.98, 6.70) | 0.49 |
| CKD stage 5Da | −8.3 (−15.93, −0.67) | 0.03 | −15.92 (−29.42, −2.43) | 0.02 |
| Kidney transplant | 1.29 (−6.68, 9.28) | 0.75 | −10.89 (−23.42, 1.64) | 0.09 |
| Height < 3rd percentile | 0.15 (−0.09, 0.39) | 0.21 | 8.14 (−0.90, 17.17) | 0.08 |
| Anemia | −4.90 (−11.46, 1.67) | 0.14 | −3.00 (−11.18, 5.19) | 0.47 |
| Number of medications | −2.06 (−0.86, 0.45) | 0.53 | 0.77 (−0.25, 1.78) | 0.14 |
| Children who left school | −0.90 (−12.31, 10.52) | 0.88 | 2.26 (−9.85, 14.38) | 0.71 |
| Not living with parents | −11.30 (−11.68, 5.37) | 0.46 | −8.94 (−31.47, 13.59) | 0.43 |
| Family income (< 10,000 THB)b | 0.38 (−9.55, 10.30) | 0.94 | −9.79 (−21.10, 1.51) | 0.09 |
| Maternal education level: high school or less | −8.19 (−15.29, −1.08) | 0.02 | −10.13 (−19.66, −0.60) | 0.04 |
| Paternal education level: high school or less | −4.02 (−12.49, 4.46) | 0.35 | 1.61 (−9.31, 12.53) | 0.77 |
Parent-reported scores: weak-to-moderate correlation with self-reported scores
(r = 0.12 – 0.42)
Abbreviations: THB: Thai baht
a Including peritoneal dialysis and hemodialysis.
b 10,000 THB is equal to approximately 290 United States dollars (USD).
Cross-sectional study
Chaichana et al. 2024
CONCLUSION: CKD stage 5D patients and patients with low maternal education had lower QoL. Regular QoL assessment is recommended for advanced CKD patients and patients with socioeconomic vulnerabilities.
